# Comparison of Protein Acetyltransferase Action of CRTAase with the Prototypes of HAT

**DOI:** 10.1155/2014/578956

**Published:** 2014-02-04

**Authors:** Prija Ponnan, Ajit Kumar, Prabhjot Singh, Prachi Gupta, Rini Joshi, Marco Gaspari, Luciano Saso, Ashok K. Prasad, Ramesh C. Rastogi, Virinder S. Parmar, Hanumantharao G. Raj

**Affiliations:** ^1^Department of Biochemistry, V.P. Chest Institute, University of Delhi, Delhi 110007, India; ^2^Department of Chemistry, University of Delhi, Delhi 110007, India; ^3^Department of Chemistry, SRM University Haryana, Plot No. 39, RGEC, Sonepat 131029, India; ^4^Dipartimento di Medicina Sperimentale e Clinica, Università di Catanzaro “Magna Graecia” Campus “S. Venuta”, Località Germaneto, 88100 Catanzaro, Italy; ^5^Department of Human Physiology and Pharmacology “Vittorio Erspamer”, Sapienza University of Rome, Piazzale Aldo Moro 5, 00185 Rome, Italy

## Abstract

Our laboratory is credited for the discovery of enzymatic acetylation of protein, a phenomenon unknown till we identified an enzyme termed acetoxy drug: protein transacetylase (TAase), catalyzing the transfer of acetyl group from polyphenolic acetates to receptor proteins (RP). Later, TAase was identified as calreticulin (CR), an endoplasmic reticulum luminal protein. CR was termed calreticulin transacetylase (CRTAase). Our persistent study revealed that CR like other families of histone acetyltransferases (HATs) such as p300, Rtt109, PCAF, and ESA1, undergoes autoacetylation. The autoacetylated CR was characterized as a stable intermediate in CRTAase catalyzed protein acetylation, and similar was the case with ESA1. The autoacetylation of CR like that of HATs was found to enhance protein-protein interaction. CR like HAT-1, CBP, and p300 mediated the acylation of RP utilizing acetyl CoA and propionyl CoA as the substrates. The similarities between CRTAase and HATs in mediating protein acylation are highlighted in this review.

## 1. Introduction

**Figure 1:**
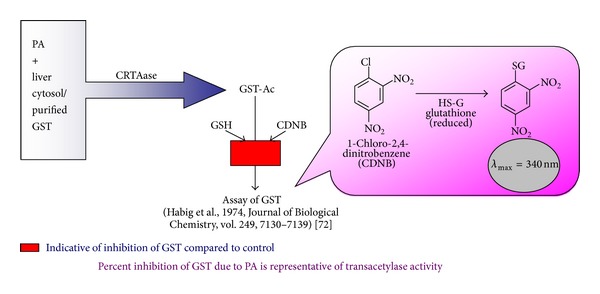
Assay for CRTAase. GST activity was assayed by the method of Habig et al., 1974 [72]. The extent of inhibition of GST activity under the conditions of assay was considered proportional to CRTAase activity. The unit of CRTAase activity was expressed in terms of percentage inhibition of GST activity [12].

Posttranslational modification of protein is a common biological mechanism that regulates protein functions by addition of functional groups such as acetate, phosphate, various lipids, and carbohydrates, changing the chemical nature of an amino acid (e.g., citrullination), or making structural changes, like the formation of disulfide bridges [1]. Protein acetylation is one of the most common covalent modifications catalyzed by a wide range of acetyltransferases [2–4]. Biological protein acetylation can be enzymatic and nonenzymatic. The enzymatic acetylation of proteins is largely confined to acetyl CoA-dependent acetylation of histones [3, 5, 6] and nonhistone proteins [2, 4, 7] by specific acetyltransferases known as HATs. The acetylation of cyclooxygenase [8] and other cellular proteins [9] by aspirin through a transacetylation reaction are examples of nonenzymatic protein acetylation independent of acetyl CoA. Arylamine N-acetyltransferases (NATs) are a class of biotransformation enzymes that catalyze acetyl group transfer from acetyl CoA, or another acetyl group donor, to the exocyclic amine of arylamines (N-acetylation) or the hydroxyl oxygen of N-hydroxylated arylamines (O-acetylation) [10]. Puzzlingly, nothing was known about the enzymatic acetylation of protein independent of acetyl CoA. Persistent work carried out in our laboratory led to the discovery of a microsomal enzyme which had the ability to acetylate certain functional proteins utilizing polyphenolic acetates (PA) as the acetyl group donor [11–14]. The protein was subsequently termed TAase. PA was found superbly effective in inhibiting hepatic microsomal cytochrome P450 (CYP)-linked mixed function oxidase by way of acetylation mediated by TAase [15]. Further an interesting observation made by us was that another enzyme in cytochrome P450 cycle, namely, NADPH cytochrome c reductase, was found to cause kinetically discernible hyperbolic activation by PA mediated by acetylation of reductase protein [11]. We then extended our studies to a cytosolic protein, glutathione S-transferase (GST), and observed that incubation of PA along with TAase resulted in the irreversible inhibition of GST and this evidence has served as an elegant assay method for TAase [12] (Figure 1). TAase catalyzed activation of nitric oxide synthase (NOS), bearing a reductase domain by PA, was a novel observation made by us [13]. Nitric oxide (NO) generated from L-arginine by NOS in the endothelium and in other cells plays a central role in several aspects of vascular biology [16]. Our investigations on the NO-related biological actions such as vasorelaxation, inhibition of ADP-induced platelet aggregation *in vitro* and *in vivo* [13, 17], activation of iNOS, and inhibition of TNF-*α* induced IL-6 in human peripheral blood mono nuclear cells (PBMC) [18] lead us to formulate the cardinal role of PA in influencing the NOS-related protein functions by way of acetylation mediated by TAase. The identity of TAase with an endoplasmic reticulum luminal protein, calreticulin (CR), was evidenced by proteomic analysis such as N-terminal sequencing, immunoreactivity with anti-calreticulin antibody, and mass spectrometry [19–21], and this novel function of CR was termed calreticulin transacetylase (CRTAase). The acetyltransferases mediated reversible protein acetylation was originally reported as a key posttranslational modification identified in nuclear proteins [3, 5, 6, 22] and then extended to cytosolic and mitochondrial proteins [2, 4, 7] by the identification and functional characterization of HATs and histone deacetylases (HDACs) in cytosol and mitochondria [23–27]. The three components of protein acetylation, namely, the receptor protein liable for acetylation of its certain lysine residues, the universal biological acetyl group donor, that is, acetyl CoA, and the protein acetyl transferase, were not hitherto conceptualized in the context of ER. Recently, a transient/reversible acetylation of a membrane bound protein *β*-site APP cleavage enzyme 1(BACE1) in the ER lumen was reported [28]. The purpose of this review is to provide an overview of the novel acetyltransferase function of ER protein CR in comparison with the well-known HATs.

## 2. Acetyltransferases

### 2.1. Histone Acetyltransferases

Acetyltransferase (or transacetylase) is a type of transferase enzyme which catalyzes the transfer of acetyl groups to the *α*-amino group of the amino-terminal residue (N-acetyltransferases) or to the *ε*-amino group of specific lysine residues (histone/factor acetyltransferases) of eukaryotic and prokaryotic proteins [29–31]. Lysine acetylation is an important covalent posttranslational modification regulating protein functions [32–35]. Unlike N^*α*^-terminal acetylation which is generally irreversible [32, 33], N^*ε*^-lysine acetylation is a reversible posttranslational process. The dynamic equilibrium of lysine acetylation is regulated by the opposing activities of acetyltransferases and deacetylases [34–36]. Several earlier reports have suggested that many lysine residues in histones are acetylated abundantly by a group of enzymes known as histone acetyltransferases (HATs) [5, 6], and the reverse reaction is catalyzed by histone deacetylases (HDACs) [35]. Furthermore, some known HATs also acetylate nonhistone proteins such as high mobility group (HMG) proteins, transcription factors, nuclear receptors [36, 37], and *α*-tubulin [36–39]. N^*ε*^-Lysine acetylation affects many protein functions, including enzymatic activity, stability, DNA binding, protein-protein interaction, and peptide-receptor recognition, and occurs on numerous and diverse proteins [5, 40]. Like HATs, HDACs could also deacetylate nonhistone proteins [41, 42]. HATs are broadly categorized into type A, located in nucleus, or type B, cytoplasmic HATs [3, 5, 40]. Based on sequence conservation within the HAT domain and substrate (histone) specificity, nuclear HATs can be grouped into four different families, Gcn5/PCAF (includes yeast Gcn5 and its human ortholog, PCAF), MYST (includes MOZ, Ybf2/Sas3, Sas2, and Tip60), p300/CBP (includes human paralogs p300 and CBP), and Rtt109 (named for regulator of Ty1 transposition gene product 109) [3, 34]. Type A HATs are involved in acetylating nucleosomal histones in chromatin and are linked to transcription [43]. However, cytoplasmic HATs such as HAT1 are involved in a housekeeping role in the cell, acetylating nascent histones in the cytoplasm during the process of chromatin assembly [43].

### 2.2. Arylamine N-Acetyltransferases

Arylamine N-acetyltransferases catalyze the N-acetylation of arylamines and hydrazines and O-acetylation of N-hydroxy-arylamines and heterocyclic amines. The enzyme follows ping-pong kinetics, with the occurrence of a covalent acetyl-enzyme intermediate at the active site cysteine using acetyl CoA or another acetyl donor, and then transfers the acetyl group to the substrate's exocyclic nitrogen (N-acetylation) or the hydroxyl oxygen (O-acetylation) [10]. N-acetylation is considered a deactivation step and O-acetylation is an activation step, resulting in the formation of reactive arylnitrenium species that can react with DNA to form adducts [44]. These reactions are important for the bioactivation of exocyclic amine containing procarcinogens and for the metabolism of some pharmaceutical drugs [44].

### 2.3. Nonenzymatic Protein Acetylation

Aspirin is known to acetylate an ER membrane protein, cyclooxygenase 1 (COX1), specifically in the hydroxyl group of Ser-530 in its active site. This nonenzymatic acetylation results in the irreversible inhibition of COX1 [45]. COX2, an isoform of COX1, is reported to get acetylated in its active site Ser-516 in the same way as it binds to Ser-530 of COX1 [45]. Some reports claim that aspirin is capable of acetylating diverse cellular proteins such as human serum albumin [46], fibrinogen [47], hemoglobin [48], endothelial NOS [49], and tumor suppressor protein p53 [50] at the N^*ε*^-lysine residues, to regulate their functions [9]. Some workers have reported the formation of platelet activating factor (PAF) from a nonspecific and nonenzymatic transfer of acetate from acetylated bovine serum albumin and apolipoproteins (acetylated by acetic anhydride) to lyso-PAF [50]. Chemical modification of cytochrome P450 reductase (CYPR) by acetic anhydride is another example of nonenzymatic protein acetylation. Acetylation of 11 lysine residues of CYPR was found responsible for lowering its  *K*_*m*_  and causing protein interaction with cytochrome P450 [51].

## 3. Novel Function of Calreticulin: Protein Acetyltransferase

### 3.1. Calreticulin: Ca^2+^-Binding Multifunctional Protein

CR is an ER luminal Ca^2+^-binding protein and a molecular chaperone of eukaryotic cells. CR is divided into three structural and functional domains [52]. The amino terminal (N-domain) is highly conserved, globular in structure and comprise eight antiparallel *β*-strands, with a single disulfide bridge. N-domain bears a carbohydrate binding site and a high capacity Zn^2+^ binding site. It is followed by proline rich P-domain that binds Ca^2+^ with high affinity [52, 53]. The structure of P-domain as predicted by NMR studies reveals that it contains an extended region stabilized by three antiparallel *β*-sheets and interacts with other chaperones (ERp57) in the lumen of the ER [54]. The carboxy terminal (C-domain) is highly acidic bearing clusters of aspartate and glutamate residues and binds Ca^2+^ with high capacity (*K*_*d*_ ~ 1-2 mM and ~25–50 mol of Ca^2+^/mol of protein) [52, 53]. CR in coordination with its paralog calnexin functions as molecular chaperones in the correct folding of nascent N-linked glycoproteins in the ER lumen [55]. Even though CR is a soluble ER protein and calnexin is an ER membrane protein, they are known to possess amino acid structural and sequence similarity. Calreticulin is a versatile protein with multifunctional properties and plays a role in a large number of pathways and biological systems including protein folding, regulation of Ca^2+^ homoeostasis, modulation of transcriptional pathways, cell adhesion, apoptosis, and embryonic development [52, 55].

### 3.2. Calreticulin Transacetylase

In our earlier investigations, we have observed that 7,8-Diacetoxy-4-methylcoumarin (DAMC), a model PA, caused the time dependant inhibition of microsomal CYP-linked MFO [15], while the hydroxy and methoxy derivatives of 4-methylcoumarin were ineffective. The fact that DAMC needed no prior oxidative metabolism by CYP to elicit the inhibition of the monooxygenases suggested that the inhibition caused by DAMC differed from the action of classical inhibitors of CYP [15, 17]. Further, 7,8-Dihydroxy-4-methylcoumarin (DHMC), the deacetylated product of DAMC [15], failed to produce the irreversible inhibition of CYP-linked MFO, indicating the cardinal role of acetyl groups of DAMC in the inhibitory action. Surprisingly, the Ca^2+^ aggregated microsomes [20] failed to catalyze the aforedescribed MFO inhibition by DAMC. These observations strongly suggested the possible involvement of an enzyme (a Ca^2+^-binding protein) in the tissue microsome capable of mediating the transfer of acetyl group from DAMC to CYP resulting in the irreversible inhibition. Such a novel enzyme utilizing several classes of polyphenolic acetates as substrates was appropriately termed acetoxy drug: protein transacetylase (TAase) [17, 20]. Incubation of liver microsome with cytosolic GST and DAMC resulted in profound inhibition of GST activity [12]; the extent of inhibition of GST was found proportional to TAase activity. Utilizing this procedure TAase could be conveniently assayed (Figure 1). TAase from the microsomes of rat liver, human placenta, and bovine lung was purified to homogeneity [19, 20, 56]. The N-terminal sequencing of the purified TAase from rat liver and human placenta was performed, and the sequences were found to show 100% identity with CR [19, 20]. The additional proof of the TAase as CR was provided by the electrophoretic mobility shift of CR on SDS gel electrophoresis in the presence of Ca^2+^. TAase was found to migrate much slower in the presence of EGTA than Ca^2+^. The apparent M.wt. of TAase shifted from 60 kDa to 55 kDa which was found consistent with Ca^2+^ dependent electrophoretic mobility shown by other Ca^2+^-binding proteins [20, 56, 57]. The identity of TAase as CR was further confirmed by immunoreactivity with anti-CR antibody.

#### 3.2.1. CRTAase Catalyzed Acetylation of Receptor Proteins

Protein acetyltransferase function of purified TAase was substantiated by mass spectrometric analysis of the modified receptor proteins. For this purpose, TAase was incubated with DAMC and GST (subunit of GST 3-3), the modified GST so obtained was subjected to detailed MALDI-TOF and LC-MS/MS. The results clearly demonstrated the acetylation of six lysine residues of the receptor protein GST 3-3 by DAMC catalyzed by buffalo liver microsomal TAase. The N-terminal six lysines Lys-51, -82, -124, -181, -191, and -210, were found acetylated [14]. An effort was also made to examine purified CRTAase catalyzed acetylation of purified NOS by DAMC which was earlier shown to irreversibly activate microsomal CYPR [11]. Neuronal NOS (nNOS) was indeed found to be acetylated as confirmed by western blot using acetylated lysine antibody and mass spectrometry. The results analyzed by nanoscale LC-MS/MS recorded 11 distinct peptides with a significant score bearing acetylated lysine residues [58]. The distribution of acetyl moieties on nNOS was in the following order: Lys-24, -33, -38, -131, and -229 of the PDZ domain; Lys-245 of the oxygenase domain; Lys-74 and -856 of the FMN binding domain; Lys-989 of the connecting domain; and Lys-1300, -1321, and -1371 of the NADPH-binding domain. During CRTAase mediated acetylation of NOS, concomitant acetylation of CR was observed. The acetylated lysines can be expected to potentiate the augmented interaction of NOS with CRTAase resulting in the remarkable activation of NOS as observed by us earlier [13, 58]. Nadler and Strobel documented the role of acetylation of lysine residues in the activation of CYPR [51]. Accordingly, the activation of NOS bearing the reductase domain by way of acetylation was substantiated [13, 51, 58].

Patel et al. have demonstrated that the interaction of CR and isoform of NOS (eNOS) enhances the electron transfer in eNOS and mimics eNOS activation in the absence of calmodulin [59]. Lysine acetylation is reported to promote protein-protein interaction [23, 24, 30]. Thus acetylation of nNOS can be expected to potentiate the interaction with CR, eventually leading to the expression of the enhanced catalytic activity of NOS.

#### 3.2.2. Autoacetylation

During the course of CRTAase catalyzed acetylation of receptor proteins by DAMC, acetylation of CR was also observed [19–21]. Such autoacetylation property of CRTAase was ratified by immunoblotting with acetylated lysine antibody and mass spectrometry [19–21]. The acetylation of lysine residues Lys-48, -62, -64, -153, and -159 in the N-domain and -206, -207, -209, and -238 in the P-domain of CRTAase, was identified by LC-MS/MS. Computational structure modeling studies were performed to probe the effect of autoacetylation of CRTAase. Accordingly, the predicted CRTAase 3D model showed that all the loop regions of both N- and P-domain bear the acetylated lysines [21]. None of the lysine residues in the C-domain was observed to be covalently modified by acetylation. The effect of this covalent modification at N-*ε*-lysine in the homology modeled structure of CR (in the absence of X-ray crystallographic structure available for CR) was analyzed by energy minimization studies. These results revealed charge neutralization of acetylated lysine residues and breaking of intermolecular H-bond with neighboring acidic residues (such as aspartate and glutamate). Thus, the protein modification by way of acetylation of lysine residues could facilitate interaction of CR with other proteins [21]. The occurrence of acetylated CR residue (Lys-206) reported in certain cases of acute myeloid leukemia due to cytogenetic abnormalities showed evidence for the CR acetylation *in vivo* [60].

#### 3.2.3. Domain Specific Protein Acetyltransferase Function of CRTAase

Calreticulin is well perceived as a multidomain protein, and we have made efforts to correlate the protein acetyltransferase function to CR domains. The CR from a blood sucking nematode, *Haemonchus contortus,* was recently characterized by producing the recombinant full length CR and its three domains [61]. The protein sequences from *H. contortus* CR (hCR) and that of CR from human have shown >70% sequence identity. So we have utilized the clones of N-, C-, and P-domains of *H. contortus* to demonstrate the role of a specific domain of the recombinant hCR in catalyzing the acetylation of RP utilizing acetoxycoumarin as the acetyl group donor. Interestingly, we have observed that the full length hCR and the recombinant P-domain possess CRTAase activity, while the N- and C-domains had no CRTAase activity. P-domain catalyzed acetylation of recombinant GST from *Schistosoma japonicum* (rGST), which have remarkable sequence and structural identity with GST3-3 (an isoform of mu class of rat GST), was analyzed by using the acetylated lysine antibody and by nanoscale LC-MS/MS, which identified the modification of several lysine residues on rGST [62].

## 4. Calreticulin as an Acetyl CoA-Linked Protein Acetyl Transferase

We made attempts to examine whether acetyl CoA, a physiological acetyl group donor for protein acetylation, is a substrate for CRTAase. The detailed kinetic studies showed that acetoxycoumarin was a better substrate than acetyl CoA for full length recombinant *H. contortus* CRTAase (rhCRTAase) as well as for hCR P-domain TAase. In this context, enzymatic acetylation of arylamine utilizing p-nitrophenylacetate as the substrate for the NATs exhibited considerably high *V*_max⁡_ (140-fold higher) as compared to acetyl CoA when used as the acetyl group donor. Acetyl CoA is a universal biological acetyl group donor possessing a high energy thioester bond between CoA and acetyl group. The log *P* values of acetyl CoA and DAMC when determined by PM3 calculation were −6.09 and 1.79, respectively, using HyperChem 8.0 [63]. This observation indicated the lipophilic character of DAMC as compared to acetyl CoA, enabling DAMC to pass through the phospholipid layer of membrane. Recent report has shown that [28] protein acetylation in ER requires a carrier (acetyl CoA transporter) mediated translocation of acetyl CoA from cytosol into the ER lumen stimulated by lipid second messenger ceramide. Thus the more lipophilic character of DAMC makes it more suitable acetyl group donor for CRTAase mediated protein acetylation in ER. Also, acetyl CoA is a rather large molecule, with a molecular mass of 810 Da whereas the molecular mass of DAMC is 276 Da.The lysine acetylation is a result of a small alteration of the amino acid side chain in the local secondary structure, it could prevent the long  *β*-mercaptoethylamine and pantothenate moiety of acetyl CoA from obtaining efficient entry into and exit from the ER lumen. It is worth noting that P-domain catalyzed acetylation of rGST as revealed by LC-MS/MS spectra resulted in the modification of several lysine residues in common, when either DAMC or acetyl CoA was used as the acetyl group donor [62].

## 5. Assessment of the Interaction of CRTAase with Acetyl Group Donor Substrates

**Figure 2:**
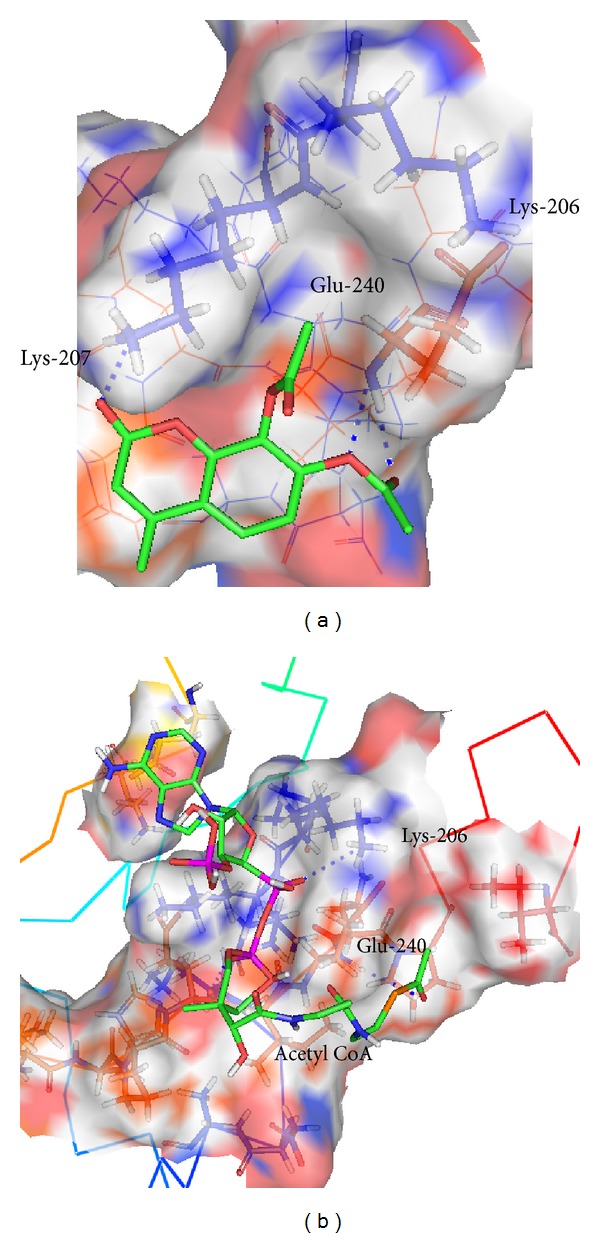
Proposed docking conformation of DAMC and acetyl CoA to the binding site (P-domain) of the 3D model structure of human CR. Basic and acidic amino acid residues are shown by blue and red colors, respectively. (a) DAMC (colored in standard atom color) forms H-bond interaction (blue dashed line) with *ε*-amino group of Lys-207 with its C-2 carbonyl oxygen and C-7 heteroatom oxygen, and carbonyl oxygen forms H-bind with backbone NH of Glu-240. (b) Carbonyl oxygen of acetyl group attached to the thiol group of CoA is hydrogen bonded with backbone NH of Glu-240, and *ε*-NH_3_ hydrogen atom of Lys-206 forms hydrogen bond with phosphate oxygen of CoA. The figure was prepared by Pymol software [73].

Blind docking of DAMC/acetyl CoA to the predicted structure of human CR using AutoDock 4.0 [64] showed that most of the docking positions of the ligand were located on the surface sites of CR. The best docking pose was obtained in the CR P-domain. The affinity of a DAMC/acetyl CoA to CR was determined by binding affinity (Δ*G*) and inhibition constant (*K*_*i*_). The best ranked docking position with lowest binding energy lies in the P-domain of CR as shown in Table 1. The putative pocket for CRTAase provides a hydrophobic environment for substrate and is rich in nonpolar residues such as Pro, Ile, and Tyr. Docking of DAMC with CRTAase showed that the complex formed H-bond between C-2 carbonyl oxygen of DAMC and -amino group of Lys-207 of CR. Furthermore, the backbone NH of Glu-240 is hydrogen bonded to the oxygen heteroatom and carbonyl oxygen at C-7 acetyl group of DAMC which is important for anchoring the C-7 carbonyl oxygen moiety of DAMC (Figure 2(a)). The deacetylation of DAMC to form DHMC and the acetylation of lysine residue of CR will take place by the nucleophilic attack by N^*ε*^-amino group of lysine residues. The side chain N^*ε*^-amino group of lysine of the catalytic nucleophile Lys-206 is located within from a distance of 3.36 Å the carbon of carbonyl moiety of DAMC in a perfect position for initiating the nucleophilic attack. Acetyl CoA was also found to bind to the same active site. The carbonyl oxygen of acetyl group attached to the thiol group of CoA forms hydrogen bond with Glu-240, and Lys-206 is hydrogen bonded with oxygen atom of phosphate moiety of CoA and is in a similar position for the nucleophilic attack (Figure 2(b)). It was observed that the binding energy of acetyl CoA with CR P-domain was 3.80 Kcal/mol lesser than that with DAMC-CR complex (Table 1), showing weaker binding of acetyl CoA with CR P-domain as compared to DAMC. Although CR P-domain was known to bind other chaperone proteins such as ERp57, the interaction with xenobiotic small molecule such as DAMC or the biomolecule such as acetyl CoA with CR was unknown. The aforementioned interaction of acetyl CoA with CR may be of considerable biochemical significance.

## 6. Computational Investigations on Acetylated GST, the Product of CRTAase Catalyzed Reaction

Analysis of the topology of the crystal structure of *S. japonicum* GST (PDB ID 1DUG) and rat mu class GST isoform, GST 3-3 (PDB ID 6GST), suggests that the acetylated residues are located in specific structural motifs (*α*-helices and *β*-turns bridging *β*-sheets) of the protein. The lysine acetylated sites were located by LC-MS/MS analysis [14, 62]. In case of *S. japonicum* GST Lys-9 and Lys-40 forming *α*-helices, Lys-27 forming *β*-turns, lied in the N-terminal  *α*/*β*  domain of the protein, Lys-78 was the only acetylated lysine residue in the linker region of *S. japonicum* GST and Lys-87, -119, -125, -131, -181, -194, and -191 (*α*-helix) and Lys-113 (*β*-turn) lied in C-terminal *α*-domain. Similarly, in rat GST 3-3 the acetylated lysine-51 (*β*-turn) and lysine-82 (*α*-helix) lied in N-terminal *α*/*β* domain and Lys-123, -181, and -191 (*α*-helices) and -210 (*β*-turn) in the C-terminal domain. It is interesting to note that the majority of acetylated lysine lies in the C-terminal domain of both the GSTs. Also, the acetylated Lys-181 and -191 are conserved in both *S. japonicum* GST and rat GST. The hydrophobicity pattern of the acetylated lysine residues shows that Lys-27, -40, -87, -131, -191, and -194 were located in the hydrophobic region, and Lys-9, -11, -78, -119, -125, and -181 lied in hydrophilic region pointing out that the stoichiometry of the acetylated lysine residues was independent of the hydrophobicity. Also, it was observed that Lys-9 and -40 were located in the proximity to the active site residues Tyr-7, Trp-8, and Trp-41. Energy minimization calculations were performed to analyze the effect of the lysine acetylation on the enzyme stability. Exploration of the energy minimized acetylated *S. japonicum* GST showed that the loss of net positive charge of acetylated lysines weakened their binding interactions with the acidic aspartate and glutamate residues. The results showed that acetylated lysines had less conformational stability and reduced tendency to form *α*-helix. As CR/CR P-domain being a stable acetylated intermediate and known to transfer acetyl group to GST, this process necessitated a protein-protein interaction. CR and in particular CR P-domain have a *β*-hairpin conformation [54], and GST having high preference for extended *β*-sheet conformation after lysine acetylation may facilitate the stronger binding of the two proteins. Also, the reduced *α*-helical tendency of Lys-9 and Lys-40 which are in the proximity of active site residues Tyr-7, Trp-8, and Trp-41 alters their binding with the substrate GSH.

## 7. Points of Similarities and Dissimilarities between CRTAase and Other Histone Acetyltransferases

### 7.1. Autoacetylated CR as Stable Intermediate

There are many features of CRTAase catalyzed protein acetylation which we have observed in common with the conventional acetyltransferases.

CRTAase was found to undergo autoacetylation during acetylation of receptor protein [19–21]. The receptor protein rGST when incubated with autoacetylated CRTAase isolated in denatured condition was found to undergo acetylation. This observation established the fact that autoacetylated CR was a stable intermediate in the protein acetyltransferase reaction [65]. It was evident from mass spectrometry that CRTAase mediated acetylation of N^*ε*^-amino group of lysine residues of RP as well in autoacetylated CR. Similar observations are reported for the different families of HATs such as p300 [66], Rtt109 [67], PCAF [68], and ESA1 [69] wherein the autoacetylated ESA1 was alone reported to be a stable intermediate as reported above. It is pertinent to point out that unlike the autoacetylated CRTAase carrying certain *ε*-NH_2_-acetylated lysine residues as described earlier, the autoacetylated ESA1 bearing a single acetylated cysteine thiol was found to function as the stable acetyl group donor. An important outcome of the acetylation of HAT is the enhancement of protein-protein interaction as evidenced in the interaction of acetylated p300 with the transcription factor p53. The autoacetylated CR was found to acquire electronegative charge due to charge neutralization of acetylated lysines [70] and facilitate the interaction of CR with RP such as GST and NOS.

### 7.2. Protein Acyltransferase Function

rhCR and P-domain were found capable of transferring propionyl group from propoxycoumarin to purified *S. japonicum* GST (rGST) [17, 65]. Hence, the calreticulin transacetylase was considered as a transacylase in general. In addition, CRTAase was also found to utilize propionyl CoA as the propionyl group donor (unpublished data). Such protein acyltransferase function was also ascribed to HATs such as HAT1, CBP, and p300 which were also found to accommodate higher acyl group donors such as propionyl CoA and butyryl CoA [71]. We have extended the investigations to explore the ability to transfer propionyl group to the receptor protein rGST by rhCRTAase utilizing propoxycoumarin as the propionyl group donor. CRTAase was reported to utilize acetyl CoA as the acetyl group donor [62].

#### 7.2.1. Acetyl CoA as a Common Substrate for CRTAase and HAT

Due to the fact that acetyl CoA being an active cellular biomolecule and is found to be a substrate for CRTAase, the possible biological implications may be envisaged. The presence of acetyl CoA in various cellular compartments such as cytosol, mitochondria, and nucleus is well recorded. However, the presence of acetyl CoA in the ER lumen is also cited [28]. On the other hand, CR although a principal resident protein of ER is reported to be localized outside ER lumen, including the cell surface, cytoplasm, and the nucleus [52]. The possible interaction of acetyl CoA with CR in the aforedescribed cellular components may be visualized although much remains to be probed in these aspects. The process of transient lysine acetylation of BACE1 in the lumen of ER is evidenced [28]. It is worth mentioning that the availability of acetyl CoA in the ER lumen utilizing ceramide as the carrier is demonstrated. This observation calls for involvement of a protein acetyltransferase in the lumen of ER and in this context. In view of our investigations, CR can play an important role in protein acetyltransferase function in ER and is well supported by other workers.

## 8. Physiological Significance of CRTAase Mediated Acetylation of Target Proteins

CRTAase catalyzed modification of CYP by acetylcoumarins leading to the inhibition of mutagen activation (e.g., formation of epoxides of aflatoxin B_1_ and benzene) has been elaborated earlier [15]. CRTAase was found to mediate the acetylation of nNOS by DAMC [13, 58]. This finding characterized polyphenolic acetates (PA) unlike the parent polyphenols, were effective enhancers of intracellular nitric oxide. Further, nitrite reductase was also profoundly activated by DAMC possibly by CRTAase catalyzed acetylation of reductase [56]. These findings implicate the usefulness of DAMC in ameliorating the conditions of hypoxia where the NOS activity is known to be compromised. The ability of DAMC was also explored to elicit the NO related biological effects such as antiplatelet action, vasorelaxation, and enhancement of vascularization in chick embryo leading to angiogenesis. These aspects of biological significance of CRTAase catalyzed acetylation of certain functional proteins have been mentioned earlier [17]. Recently, we have reported that DAMC could modulate tumor necrosis factor-alpha induced activation of iNOS [17] leading to elevated cellular NO levels. Also, DAMC inhibits TNF-*α* mediated release of IL-6 probably by inhibiting TNF-*α* stimulated activation of some specific kinases (MAP kinases), thus contributing to the anti-inflammatory effect of PA [18]. Further, protein kinase C (PKC) was inhibited probably by CRTAase mediated acetylation by DAMC. PKC activity was reported to increase in the case of chronic obstructive pulmonary disease (COPD); also an imbalance of iNOS/eNOS levels were observed in such cases. The fact that DAMC causes the modulation of NOS activity (both eNOS and iNOS), as well as inhibition of PKC. Hence, DAMC can be thought to restore the underlying biochemical changes of COPD (unpublished data).

## 9. Conclusion

Acetylation is one of the prominent covalent modifications. Acetylation of proteins by acetyl CoA catalyzed by specific acetyltransferases is well documented. Nonenzymatic protein acetylation by acetylated xenobiotics such as aspirin is also known. Acetylation of proteins independent of acetyl CoA was hitherto unknown. Our persistent investigations for more than one decade embodied in this review culminated in the understanding of the acetyltransferase function of calreticulin utilizing PA as the acetyl group donor. The points of agreement and differences between CRTAase and prototype acetyltransferases in particular are highlighted. It is now evident that the protein acetyltransferase property has contributed to the novel functions of CR not reported earlier.

## Figures and Tables

**Table 1:** AutoDock free energy binding (Δ*G*) and estimated inhibition constants (*K*_i_) of the CR substrates (temperature 298.15 K).

Compounds	Binding energy Δ*G* (Kcal/mol)	Inhibition constant *K*_i_ (*μ*M)
DAMC	−4.79	102
Acetyl CoA	−0.95	225
